# GSP4PDB: a web tool to visualize, search and explore protein-ligand structural patterns

**DOI:** 10.1186/s12859-020-3352-x

**Published:** 2020-03-11

**Authors:** Renzo Angles, Mauricio Arenas-Salinas, Roberto García, Jose Antonio Reyes-Suarez, Ehmke Pohl

**Affiliations:** 1 Department of Computer Science, Universidad de Talca, Camino Los Niches Km 1, Curicó, Chile; 2 Millennium Institute for Foundational Research on Data, Santiago, Chile; 3 Centro de Bioinformática y Simulación Molecular, Universidad de Talca, Talca, Chile; 4 Faculty of Engineering, Universidad de Talca, Camino Los Niches Km 1, Curicó, Chile; 5 Department of Chemistry, Durham University, Durham, DH1 3LE United Kingdom; 6 Department of Biosciences, Durham University, Durham, DH1 3LE United Kingdom

**Keywords:** PDB, Protein-ligand interaction, Structural patterns, Big data

## Abstract

**Background:**

In the field of protein engineering and biotechnology, the discovery and characterization of structural patterns is highly relevant as these patterns can give fundamental insights into protein-ligand interaction and protein function. This paper presents GSP4PDB, a bioinformatics web tool that enables the user to visualize, search and explore protein-ligand structural patterns within the entire Protein Data Bank.

**Results:**

We introduce the notion of graph-based structural pattern (GSP) as an abstract model for representing protein-ligand interactions. A GSP is a graph where the nodes represent entities of the protein-ligand complex (amino acids and ligands) and the edges represent structural relationships (e.g. distances ligand - amino acid). The novel feature of GSP4PDB is a simple and intuitive graphical interface where the user can “draw” a GSP and execute its search in a relational database containing the structural data of each PDB entry. The results of the search are displayed using the same graph-based representation of the pattern. The user can further explore and analyse the results using a wide range of filters, or download their related information for external post-processing and analysis.

**Conclusions:**

GSP4PDB is a user-friendly and efficient application to search and discover new patterns of protein-ligand interaction.

## Background

In the context of protein engineering and biotechnology, structural patterns are three-dimensional structures that occur in biological molecules, such as proteins or nucleid acid, and are key to understand their functionality [1]. The discovery and characterization of structural patterns is an important research topic as it can give fundamental insight into protein function, and represents an important tool to decipher the function of novel proteins [2, 3].

We concentrate our interest on structural patterns representing protein-ligand interactions [4]. Ligands are small molecules (such as ATP, drug and metal) that can interact, bind and control the biological function of proteins. Finding common binding sites in weakly related proteins may lead to the discovery of new protein functions and to novel ways of drug discovery [5].

The study of the specific interaction of a protein with a ligand is an active research field because of the implications this has in the overall understanding of the structure and function of proteins, and in particular in the fast-growing area of rational drug design [6]. Particularly, structure-based drug design/discovery is one of the computer-aided methods by which novel drugs are designed or discovered based on the knowledge of 3D structures of the relevant specific targets [7–9].

The importance of structural patterns can be exemplified by the Zinc finger motif that is widely found in DNA binding proteins including many eukaryotic transcription factors [10, 11]. Although proteins containing this motif perform a wide range of functions in various cellular processes, they all rely in the same underlying structural pattern [12].

To the best of our knowledge, there is no standard way to represent and search protein-ligand structural patterns (like the Zinc Finger motif) in structure databases. One way to represent patterns is to use a textual format. For instance, PROSITE defines the PA line notation [13] which allows to represent the classical zinc finger pattern as the text string C-x(2,4)-C-x(12)-H-x(2,6)-H. A similar notation can be found in several articles [11, 14, 15]: CX _2−4_CX_12_HX _2−6_H. Note that both notations are based on primary structure alone, so they are restricted to express sequence patterns (i.e. a sequence of amino acid symbols).

There are only limited tools available to search and analyse these patterns in the sequence and structural databases. They rely on the input of simple numerical values and the results are usually represented as tables or statistical charts such as histograms. Therefore, the searches are complicated and cumbersome to visualise making the exploration of structural pattern difficult and non-intuitive. Different types of visual representations used by current tools and systems are described in the section about Related Work.

Considering the problems identified above, we propose a graph-based model for representing structural patterns. Specifically, any protein-ligand structural pattern can be described as a graph whose nodes describe amino acids or ligands, and the edges represent their relationships. Based on this model we have developed GSP4PDB, a web application that enables the non-expert user to design, search and analyse protein-ligand structural patterns inside the Protein Data Bank (PDB) [16].

### Protein-ligand structural patterns

Proteins are structurally complex and functionally sophisticated molecules, whose existence is essential to all forms of life with their wide ranging roles in all organisms [17].

There are four levels of organization in the structure of a protein. The *primary structure* refers to the sequence of amino acids, which are linked by peptide bonds to form polypeptide chains. Polypeptide chains can fold into regular structures such as the alpha helices and beta sheets. These substructures, stabilized by regular H-bonding between the main chain atoms, conforms the *secondary structure* of the protein. *Tertiary structure* refers to the full three-dimensional organization of one polypeptide chain. Finally, if a particular protein is formed by more than one polypeptide chain, the complete structure is designated as the *quaternary structure* [18].

The notion of *structural pattern* is used to describe a three-dimensional “structure” or “shape” of motifs such as ligand binding sites in the protein [19]. The same structural pattern can occur in a group of proteins with a given frequency and satisfying specific criteria (e.g. atomic distance, composition, connectivity, etc.). There are several types of structural patterns, but we concentrate on those representing protein-ligand interactions [4].

We define a *protein-ligand structural pattern* as the combination of a ligand and a group of amino acids, whose three-dimensional distribution could be determined by different types of relationships, including the distance between two amino acids, the distance between an amino acid and the ligand, and the order or precedence (in the sequence) of an amino acid with respect to other amino acid.

**Fig. 1:**
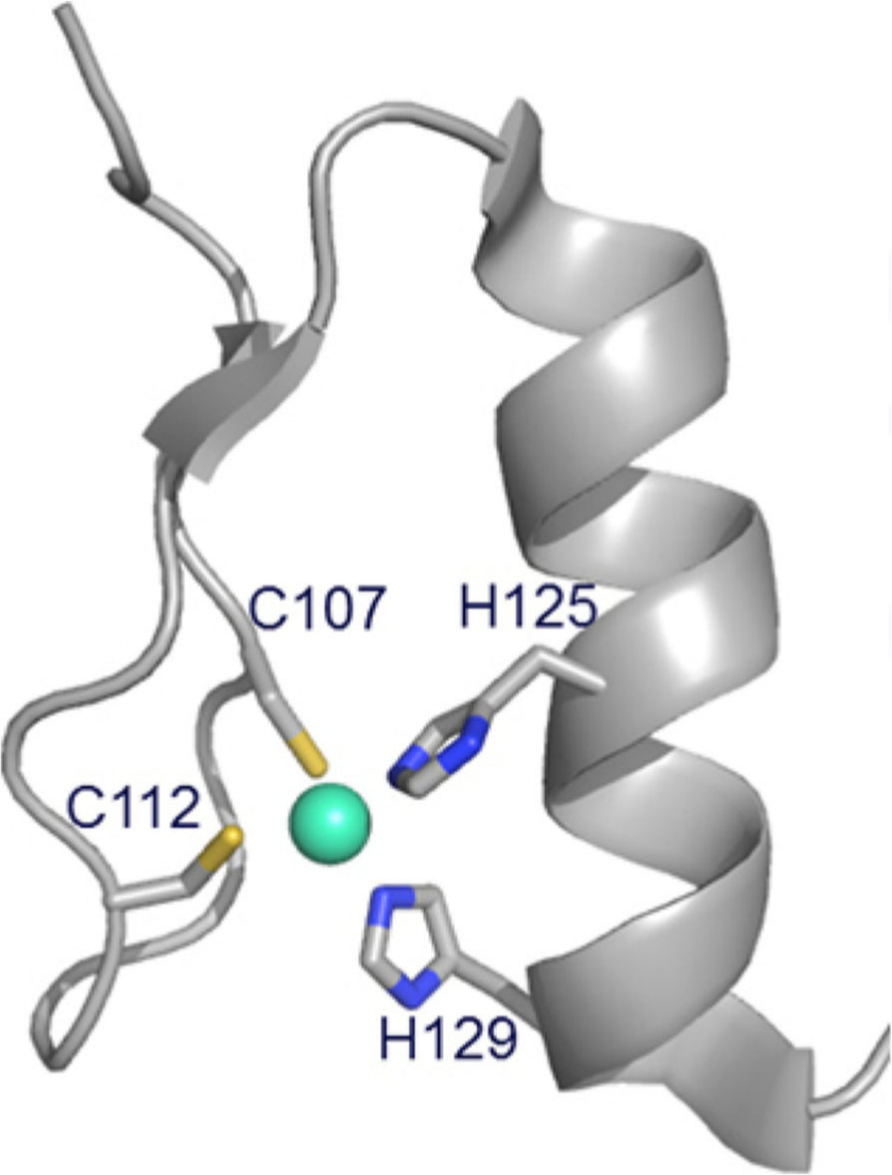
Three-dimensional representation of the Zinc finger pattern characteristic of the Cys2His2 type. Four residues (Cys107, Cys112, His125 and His129) coordinate to the zinc ion (cyan ball)

For instance, a Cys2His2 zinc finger [14] is a protein-ligand structural pattern where one Zn2+ ion (the ligand) is tetrahedrally coordinated by cysteine and histidine residues (the amino acids). Figure 1 shows a three-dimensional representation of the Cys2His2 zinc finger [11].

**Fig. 2:**
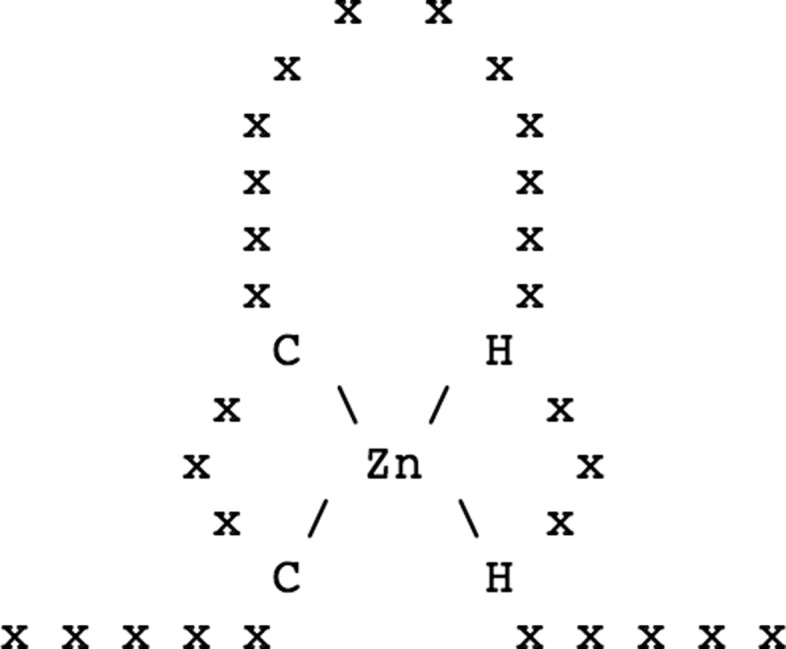
A schematic representation of a Zinc Finger found in PROSITE

A schematic representation of the Zn2+ binding site of a zinc finger is shown in Fig. 2. According to the PROSITE notation [20], the above pattern can be represented with the text expression x(5)-C-x(3)-C-x(12)-H-x(3)-H-x(5). Importantly, this representation refers to the primary structure only.

On the one hand, the schematic representation is sufficient to show the protein-ligand interaction (including some structural details). On the other hand, the textual representation provides a simple syntax to describe the structure of the sub-sequence participating of the binding site. However, the textual description does not contain any detail such as distances and geometry. In addition, the single-letter amino-acid representation is mainly used by bioinformaticians and hence limits its general use.

In order to circumvent the limitations described above, we propose the use of graphs as a simple and intuitive way to represent and visualize structural patterns.

### Graph-based structural patterns

**Fig. 3:**
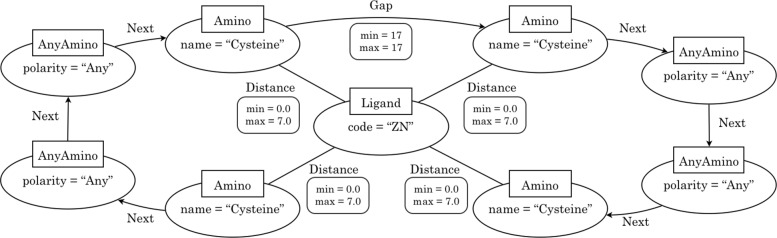
Graph-based structural pattern for a GATA-type zinc finger

In general terms, a *graph-based structural pattern (GSP)* is a graph where the nodes represent protein’s components (i.e. amino acids and ligands) and the edges represent structural relationships (e.g. distance between amino acids). For instance, Fig. 3 shows a GSP that corresponds to a Zn2+ binding site in a GATA-type zinc finger. GATA factors coordinate cellular maturation with proliferation arrest and cell survival, therefore they play important roles in human cancers [21].

The textual representation of a GATA zinc finger [22] is given in PROSITE by the expression C-x(2)-C-x(17)-C-x(2)-C. This textual representation denotes that a single zinc ion is coordinated by 4 cysteine residues such that, between the first and the second cysteine there are two amino acids of any kind, 17 amino acids between the second and third, and again two amino acids of any kind between the third and the fourth.

Formally, a *graph-based structural pattern* is a labeled property graph, i.e. a labeled graph where nodes and edges can contain key-value pairs representing their properties (or attributes). Four types of nodes are allowed: amino-acid-nodes, any-amino-acid-nodes, ligand-nodes and any-ligand-nodes. Additionally, nodes can be connected by three types of edges: distance-edges, next-edges and gap-edges.

Nodes are drawn as ellipses whose label (inside a square) determine their type. An *amino-acid-node* represents a specific residue, whose name is defined by the property *name*. An *any-amino-acid-node* represents the occurrence of any amino acid (as a variable). Each any-amino-acid-node includes the property “polarity”, whose value can be “any”, “non-polar”, “polar uncharged”, “positively charged” or “negatively charged”. A *ligand-node* represent the ligand of the pattern, whose 3-letter code is defined by the property “code”. An *any-ligand-node* represents any ligand (similar to an any-amino-acid-node).

Edges are labeled with their type (distance, next or gap), and can be directed (next-edges and gap-edges) or undirected (distance-edges). A *distance-edge* is an undirected edge which represents the distance relationship between two amino acids, or between an amino acid and the ligand. A distance-edge includes the properties *min* and *max*, which allow to define the minimum and maximum distance expressed in Angstroms (a distance range between 0.5Å and 7Å). Given two amino acids *X* and *Y* (specific or any), a *next-edge* allows to specify that *X* follows *Y* in the sequence, and a *gap-edge* represents the occurrence of a given number of amino acids in the sequence between *X* and *Y*. The number of amino acids is defined by the properties *min* and *max*, such that *min*>0,*max*≥*min*, and *max*=∗ represents an undefined number.

Note that our graph-based representation is a simple and intuitive way to describe and recognize the two-dimensional structure of a protein-ligand pattern. Importantly, the model proposed here could be extended to represent other types of structural patterns.

## Implementation

Using the graph-based structural patterns, we have developed GSP4PDB, a bioinformatics tool that allows the user to analyse protein-ligand interactions within the entire protein data bank. GSP4PDB is formed by three main elements: gsp4pdb-parser, a java tool which extracts and processes data from PDB coordinate files; a relational database (PostgreSQL) which is used to store and manage protein data; and a web application which provides a graphical interface to visualize, search and explore graph-based structural patterns.

### Protein data extraction and pre-processing

GSP4PDB was designed to work using data obtained from the Protein Data Bank (PDB) [16]. Therefore, we have developed gsp4pdb-parser, a command-line java application which allows to process PDB files and export the data to a relational database system.

We use rsync to maintain a local copy of the entire protein data bank. So, each time gsp4pdb-parser is executed, the relational database is updated with the latest proteins released in the main PDB repository. The current version of gsp4pdb-parser is restricted to process files encoded using the PDB format (*.pdb, *.ent or *.ent.gz).

To execute gsp4pdb-parser, the user must specify a local directory where the PDB files are stored. Hence, gsp4pdb-parser explores the directory (recursively) and prepares (internally) a list of available PDB files. Such list is filtered according to the proteins available in the relational database, whose corresponding files were processed previously. Optionally, the user can specify a list of protein IDs to be processed.

For each file of the filtered list, gsp4pdb-parser reads the file using biojava [23] and creates an object model of the protein. The main classes of the model are Protein, SChain, Aminoacid, AminoStandard, AminoStandardList, Ligand, AtomAmino, AtomLigand and Distance. Although a protein can contain many chains, at the moment only the chain with the largest number of amino acids is being processed for simplicity.

Note that a PDB file does not contain explicit information about specific atomic distances. In order to improve the performance of the system, which relies on complex join operations for the relational database, some distances are pre-computed in the initial phase.

Therefore, during the construction of the object model two distance measures are pre-computed: distance amino-amino and distance ligand-amino. The distance between two amino acids *A* and *A*^′^, is calculated as the minimum distance between each pair of atoms (*a*_*i*_,*a*_*j*_) such that *a*_*i*_∈*A* and *a*_*j*_∈*A*^′^ (i.e. we compute the distance between each pair of atoms of *A* and *A*^′^). A similar approach is applied to determine the distance between a ligand *L* and an amino acid *A*. Distances greater than 7.0Å are not considered as we assume that there is no interaction between the atoms. Additionally, we define the class NextAminoAmino to represent the sort between each pair of amino acids in the chain.

After the object model of the protein is constructed, gsp4pdb-parser loads the data to the relational database system using a single bulk of SQL instructions. Next we describe the relational model used to store and manage the protein data.

### Protein data storage

**Fig. 4:**
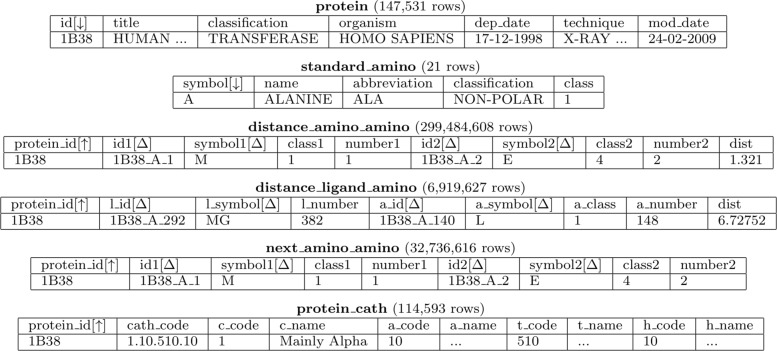
Structure of the relational database used by GSP4PDB. For each table we show table’s name, rows number, attribute and a sample data row. Primary keys and foreign keys are marked with [*↓*] and [*↑*] respectively. Indexed attributes are marked with [*Δ*]

GSP4PDB uses a PostgreSQL database system (version 9.4) for storing and managing protein data obtained from the PDB repository. The current database contains information of 147,531 proteins (latest synchronization on February 1, 2019). The database is formed by the relational tables listed in Fig. 4.

The table “protein” contains general information about each protein. Information about the twenty standard amino acids, plus an “undefined” amino acid, is stored in the table “standard_amino”. Most of the data rows in the database corresponds to the tables “distance_amino_amino” (distances between each pair of amino acids), “distance_ligand_amino” (distances between ligands and amino acids) and “next_amino_amino (sequential relationship between amino acids). Recall that the information of these tables is not provided (explicitly) by PDB, so it is computed during the pre-processing phase. The table protein_cath contains information about the CATH classification [24] of 114,593 proteins.

Figure 4 also shows the primary keys (attributes that identify rows in a table) and the foreign keys (attributes that refer a primary key in other table) in the database. Note that the attributes named “id” have been designed to describe data provenance. For instance, the atom_amino having id = “1B38_A_1_4” describes the atom number 4, that belongs to the amino acid number 1, of the chain “A”, in the protein “1B38”.

Note that the database contains duplicated data in several tables (i.e. there is data redundancy). This denormalized design was selected in order to improve query computation and, consequently, to reduce the response time of the database system. The efficiency of the systems is also supported by the inclusion of 12 B-tree indexes (indicated in Fig. 4 with the symbol *Δ*), plus the unique indexes created automatically for primary keys. This is a stable configuration which we expect to improve in the future.

### Web user interface

**Fig. 5:**
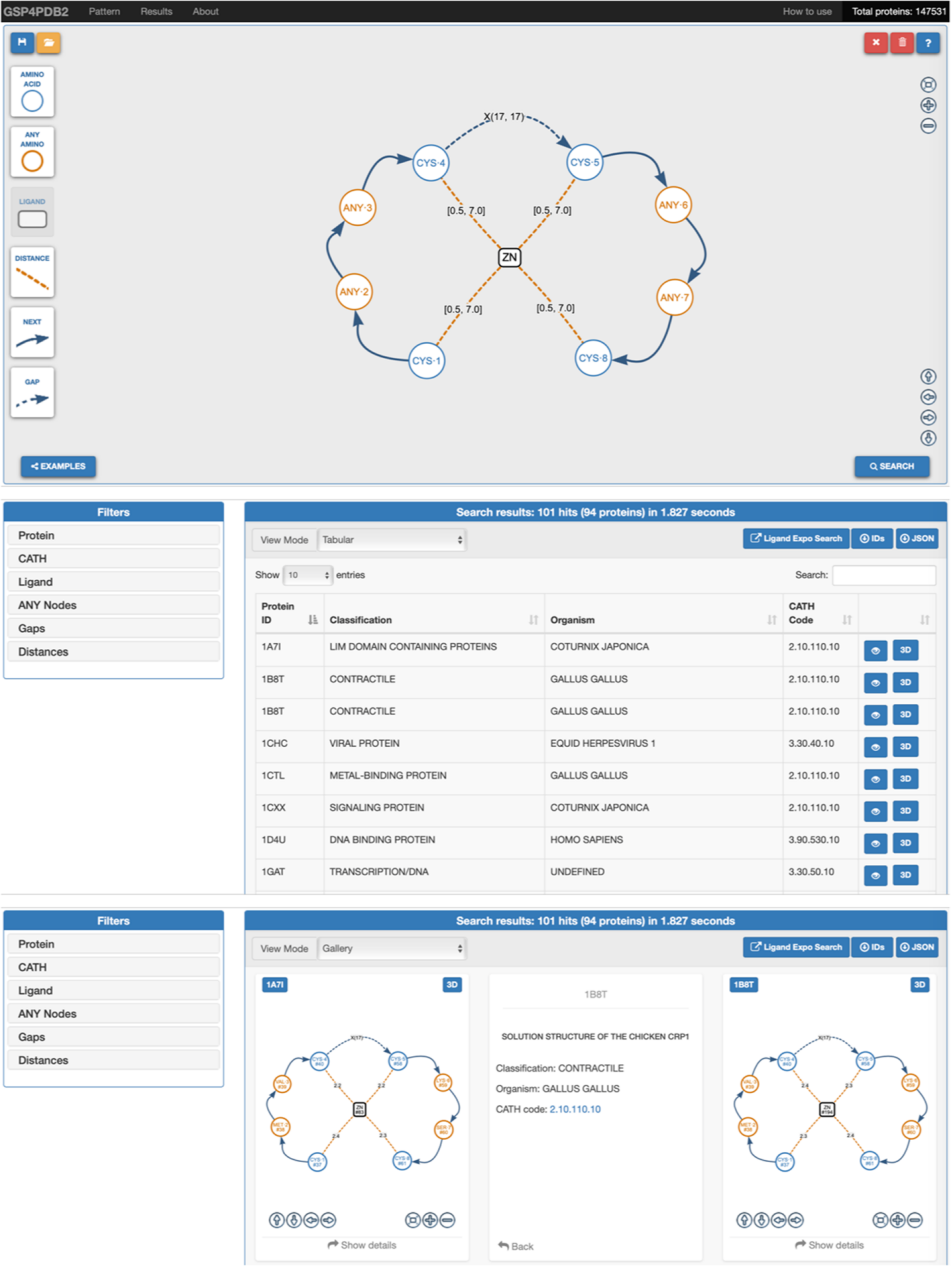
Components of the GSP4PDB web interface: (Top) Navigation bar and Design area; (Middle) Output area in Tabular view mode; (Bottom) Output area in Gallery view mode

GSP4PDB includes an intuitive Web interface which allows to create a protein-ligand structural pattern, search the pattern in the relational database, and explore the search results using tabular and graphical representations. The web interface can be divided in three main components (see Fig. 5): the Navigator Bar, the Design Area and the Output Area.

The *Navigation Bar* allows to navigate among the main elements of the interface. This bar shows the number of database entries, and includes a button to display a “How to use” popup containing a short description about the use of the tool.

The *Design Area* allows the user to “draw” a GSP by using drag-and-drop of buttons associated to the types of nodes and edges allowed in a GSP (the LIGAND button allows to create both ligand-nodes and any-ligand-nodes). On the right-hand side of the Design Area, there are buttons to move the pattern, delete elements, or clean the design space. There is also a “help” button which allows to display informative text (tooltips) over the buttons of the interface.

The Design Area shown in Fig. 5 contains a GSP which is equivalent to the one presented in Fig. 3. Each amino-acid-node is labeled with the 3-letter code of the corresponding amino acid, followed by its node identifier (e.g. CYS-1). Similarly, an any-amino-acid-node is labeled with the ANY prefix and the corresponding node identifier. Each distance-edge is represented as a dashed line and is labeled with a distance range (where [0.5,7.0] is the default assignment). Next-edges are represented as traditional arrows, and gap-edges are shown as dashed-arrows labeled with a gap range of the form X(min,max). The properties for nodes and edges can be changed by doing double-click on them.

Importantly any GSP can be saved and uploaded later again to allow the user to modify and optimize previous searches. In both cases, the GSP is managed as a JSON file having a special structure. In a similar way, the user is able to upload a GSP sample by clicking the “Examples” button. The *Output Area* shows the results of searching the GSP in the database, and provides filters that allow to further explore and analyse the results. The results can be viewed in Tabular or Gallery mode. Each row in the Tabular view mode shows information about a protein containing the pattern, a button to “see” more information about the solution (including a graph-based representation), and a “3D” button which allows to visualize the binding site in a JSmol popup.

In the Gallery view mode the solutions are shown as a collection of “cards”. Each card contains the PDB ID of one matched protein, a graph-based representation of the binding site (similar to the input GSP), and a “Show details“ button that flips the card to see additional information about the solution.

GSP4PDB includes a set of filters (or facets) that can be used to analyse the results. The filters are organized in six groups: “Protein” allows to filter the results by PDB ID, Classification and Organism; “CATH’ includes filters to explore the CATH structural hierarchy (i.e. Class, Architecture, Topology/fold and Homologous superfamily) [25]; “Ligand” is active when an any-ligand node is used; “ANY Nodes”, “Gaps” and “Distance” include a filter for each occurrence of an any-node, a gap-node or a next-edge. In order to support further off-line analysis, the user is also able to download the list of protein IDs or the solutions in their JSON encoding.

### From graph patterns to sQL queries

Recall that GSP4PDB stores the protein data in a relational database (in this case, PostgreSQL). Hence, the simplest way to query the database is to use the SQL query language.

In this section we present a brief description of the method to transform a graph-based structural pattern into a SQL query expression. In general terms, the method generates a SQL query expression for each node-edge-node structure in the graph pattern. The final SQL query, expressing the complete graph pattern, is the compositions of all the sub-expressions.

The method defines transformations for the following node-edge-node structures:

- Ligand ⋯ Distance ⋯ Amino
- Ligand ⋯ Distance ⋯ ANY-amino
- ANY Ligand ⋯ Distance ⋯ Amino
- ANY Ligand ⋯ Distance ⋯ ANY-Amino
- Amino — Distance — Amino
- Amino — Distance — ANY-amino
- ANY-amino — Distance — ANY-amino
- Amino — Next → Amino
- Amino — Next → ANY-amino
- ANYa-mino — Next → Amino
- ANY-amino — Next → ANY-amino
- Amino — Gap → Amino
- Amino — Gap → ANY-amino
- ANY-amino — Gap → Amino
- ANY-amino — Gap → ANY-amino

For instance, the SQL query corresponding to a Ligand-distance-Amino structure (case 1) is the following:


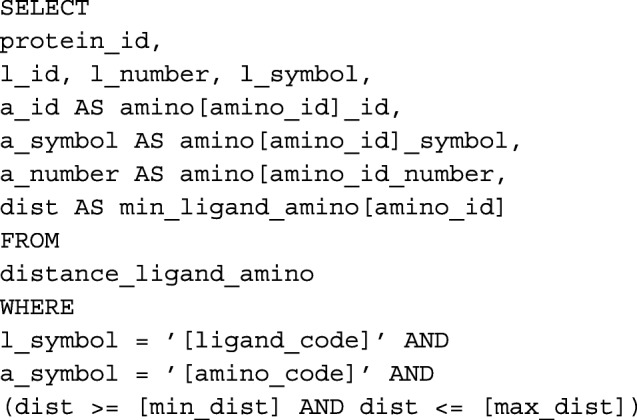


The above SQL expression is a template for querying a distance relationship between a ligand and an amino acid. Note that the parameters of the template, represented as square brackets, should be replaced with values from the graph pattern in order to obtain the final SQL expression. For the sake of space, we do not present the rest of transformations. We refer the reader to the complete documentation of GSP4PDB which is available at https://structuralbio.utalca.cl/gsp4pdb/.

## Results

The utility of GSP4PDB was evaluated through the zinc finger domain. There are several groups for this domain that differ in the type of amino acids present in the pattern and the structural characteristics [12].

### Case study 1: visualization of structural patterns

In order to evaluate GSP4PDB in terms of its features to design and visualize structural patterns, we have selected the Cys2His2 motif, a well-characterized class of zinc fingers which is related to a large number of regulatory proteins in mammals [14].

**Fig. 6:**
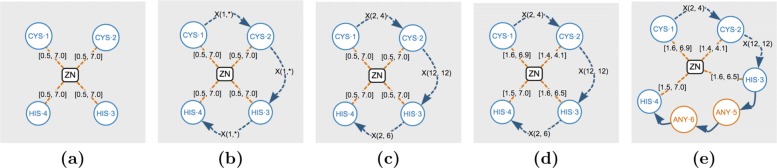
Test patterns related to the Cys2His2 zinc finger. Search results: (**a**) 55,740 hits in 2,407 proteins; (**b**) 2,354 hits in 1,006 proteins; (**c**) and (**d**) 630 hits in 343 proteins; (**e**) 4 hits in 4 proteins

Figure 6 shows five patterns which were used to identify different Zinc Finger motifs in the Protein Data Bank. Pattern (a) shows a general GSP formed by two cysteines, two histidines, the zinc ligand, and four distance relations (all of them configured with a default range between 0.5 and 7.0Å). The search of pattern (a) results in 55,740 hits (occurrences), distributed in 2,407 proteins.

The large number of results in pattern (a) is due to the fact that no sequence order of the amino acids was given. Hence, for each result there will be several “mirror” results. This issue can be solved easily by including GAP edges, as they introduce a sequential restriction to the pattern. This is reflected in pattern (b), where the addition of three gap-edges reduces the number of hits to 2,354.

Note that all the gaps in pattern (b) have a range X(1,*), i.e. one or more amino acids in the gap. Following the results presented in [14], where the structure of the Cys2His2 zinc finger is described with the expression CX _2−4_CX_12_HX _2−6_H, the general pattern can be restricted to the one shown in example (c). Recall that all the distance-edges were configured with the default range, i.e. [0.5, 7.0]. In order to find the specific range for each distance, we can make use of filters. This allows us to define the ranges shown in example (d). Note that both patterns (c) and (d) have 630 hits. Finally, we can use any-amino-acid-nodes to search for specific sub-sequences inside a gap. For instance, assume we are interested in exploring subsequences of large 2 between the two histidines. This can be done by replacing the third gap by two any-amino-acid-nodes, as shown in example (e). The four results of this latest pattern can be further explored in a visual way or by using the corresponding filters. In both cases, we discovered that the subsequences ILE-ARG and LYS-ASP occur only once while GLU-ILE twice.

GSP4PDB filters allow to group results and calculate summarized data. Table 1 shows the sum of hits for specific groups of keywords found in the classification of the proteins containing the pattern (d). This summary shows that 9 results contain the keyword “ZINC FINGER” in their classification. It is important to note that as the classifications given in the PDB entry is often incomplete, any simple text search is not able to identify the majority of these Zinc fingers in the PDB. We expect to further optimize the pre-processing of the classification values in the future versions of GSP4PDB.

**Table 1:** Example of summarized data calculated over the results of the Cys2His2 pattern shown in Fig. 6d

Keywords	Hits
TRANSCRIPTION (188)	409
TRANSCRIPTION/DNA (185)	
TRANSCRIPTION REGULATOR/DNA (14)	
TRANSCRIPTION, METAL BINDING PROTEIN (2)	
TRANSCRIPTION FACTOR/DNA (9)	
TRANSCRIPTION REGULATION (7)	
TRANSCRIPTION REGULATOR (2)	
TRANSCRIPTION/RNA (2)	
DNA BINDING PROTEIN (23)	109
DNA-BINDING PROTEIN (4)	
DNA BINDING PROTEIN/DNA (79)	
DNA BINDING PROTEIN/RNA/DNA (3)	
METAL BINDING PROTEIN (21)	39
METAL BINDING PROTEIN/DNA (10)	
DNA/METAL BINDING PROTEIN (3)	
TRANSCRIPTION, METAL BINDING PROTEIN (2)	
NUCLEAR PROTEIN/METAL BINDING PROTEIN (3)	
TRANSFERASE (2)	21
TRANSFERASE/DNA (19)	
GENE REGULATION (10)	15
GENE REGULATION/DNA (5)	
ZINC FINGER (3)	9
ZINC FINGER DNA BINDING DOMAIN (6)	
RNA BINDING PROTEIN (6)	9
RNA-BINDING PROTEIN/RNA (2)	
RNA BINDING PROTEIN/RNA (1)	
HYDROLASE/DNA (1)	6
HYDROLASE/DNA/RNA (5)	
UNKNOWN FUNCTION	5
PROTEIN BINDING (2)	2
CELL CYCLE (2)	2
TRANSLATION REGULATOR (1)	2
TRANSLATION (1)	
SPLICING (2)	2
LIGASE (1)	1
VIRUS (1)	1

Importantly, GSP4PDB can be used to identify the functions of proteins that have not been annotated previously. Our analysis (Table 1) showed that there are 5 proteins in PDB having the Cys2His2 motif, and annotated with “Unknown Function” in their keyword property. Therefore, we could assume that if these proteins possess the domain of Zinc Finger, they are likely to be associated with DNA binding.

Recall that the GSP4PDB database contains CATH information as a complement to the data obtained from PDB. Table 2 shows information about the CATH classifications related to the Cys2His2 motif. For instance, there were 293 hits for the hierarchical level 3.30.160.60 corresponding to the “classical” Zinc finger motif.

**Table 2:** CATH information about the solutions of the Cys2His2 pattern shown in Fig. 6d

Class	Architecture	Topology/fold	Homologous superfamily	Hits	CATH code description
3	-	-	-	300	Alpha Beta
3	30	-	-	300	2-Layer Sandwich
3	30	160	-	293	Double Stranded RNA Binding Domain
3	30	160	60	293	Classic Zinc Finger
3	30	428	-	4	HIT family, subunit A
3	30	428	10	4	HIT-like
3	30	40	-	3	Herpes Virus-1
3	30	40	130	2	Herpes Virus-1
3	30	40	200	1	Herpes Virus-1
2	-	-	-	3	Mainly Beta
2	170	-	-	1	Beta Complex
2	170	270	-	1	Beta-clip-like
2	170	270	10	1	SET domain
2	60	-	-	1	Sandwich
2	60	40	-	1	Immunoglobulin-like
2	60	40	10	1	Immunoglobulins
2	30	-	-	1	Roll
2	30	170	-	1	Ribosomal Protein L24e; Chain: T;
2	30	170	10	1	Ribosomal Protein L24e; Chain: T;
1	-	-	-	1	Mainly Alpha
1	10	-	-	1	Orthogonal bundle
1	10	10	-	1	Arc Repressor Mutant, subunit A
1	10	10	790	1	Arc Repressor Mutant, subunit A
No value	No value	No value	No value	326	

GSP4PDB also facilitates the analysis of the interaction distances between the ligand and the different amino acids that make up the pattern, through the analysis of the data that are downloaded from the JSON file. An example of this is shown in Table 3 where the values of the average distances of the interactions between the Zn and the amino acids grouped by CATH code are shown.

**Table 3:** Average distances for the solutions of the Cys2His2 pattern shown in Fig. 6d

CATH code	Average distance				Hits
	C _1_-Zn	C _2_-Zn	H _1_-Zn	H _2_-Zn	
3.30.160.60	2.32	2.28	2.09	2.12	293
3.30.428.10	2.35	2.23	2.02	2.04	4
3.30.40.130	2.13	2.13	1.99	6.96	2
3.30.40.200	2.20	2.38	2.10	2.18	1
2.170.270.10	2.29	2.28	2.12	2.12	1
2.60.40.10	2.61	2.51	2.39	2.41	1
2.30.170.10	4.39	4.01	5.11	6.49	1
1.10.10.790	2.52	2.66	2.36	2.38	1
No value	2.33	2.25	2.07	2.08	326

This table shows the average distances for interactions between ligand Zn and the amino acids (Cys _1_, Cys _2_, His _1_ and His _2_), grouped by CATH code

This analysis clearly shows that class 2.30.170.10, which corresponds to a ribosomal protein, is not a genuine Zn2+ binding as all distances are much larger than expected. If the user were interested in accurate average bond distances the search can easily be repeated with a smaller range such as distances between 1.5 and 3Å.

### Case study 2: search of structural patterns

**Fig. 7:**
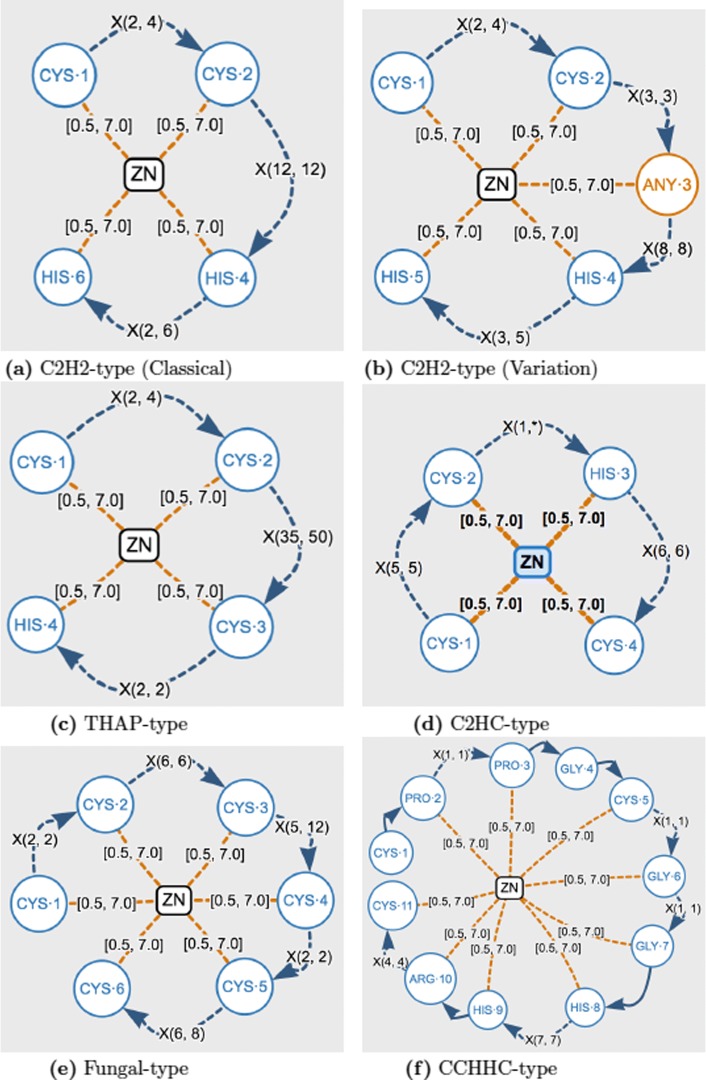
Graph-based structural patterns for six classes of zinc fingers

In order to illustrate the use of GSP4PDB to represent and search different types of protein-ligand structural patterns, we selected six classes of zinc fingers (C2H2 classical [14], C2H2 variation [26], THAP [27], C2HC [28], Fungal, CCHHC [29]). The corresponding graph-based structural patterns are shown in Fig. 7, and the results of their search are shown in Table 4.

**Table 4:** Six classes of zinc fingers used in case study 2 (C2H2 classical, C2H2 variation, THAP, C2HC, Fungal, CCHHC)

Zinc Class	Textual Pattern (PROSITE convention)	Hits	Time (s)
C2H2-c	C-x(2,4)-C-x(12)-H-x(2,6)-H	630	1.72
C2H2-v	C-x(2,4)-C-x(3)-[LIVMFYWC]-x(8)-H-x(3,5)-H	554	2.89
THAP	C-x(2,4)-C-x(35,50)-C-x(2)-H	36	1.64
C2HC	C-x(5)-C-x(n)-H-x(6)-C	6	0.83
Fungal	C-x(2)-C-x(6)-C-x(5,12)-C-x(2)-C-x(6,8)-C	28	0.87
CCHHC	C-P-x(1)-P-G-C-x(1)-G-x(1)-G-H-x(7)-H-R-x(4)-C	1	1.14

For each pattern we present the number of results (hits) and the computation time (in seconds)

Figure 7a shows a simple pattern that describes the structure of the classical Cys2His2 zinc finger [14]. A variation of this class is presented in Fig. 7b, where the any-amino-acid-node ANY ·3 is used to represent a special sub-sequence between the amino acids CYS ·2 and HIS ·4. Figure 7c shows a pattern containing a large-range gap-edge, i.e. X(35,50). Similarly, the pattern shown in Fig. 7d contains the gap-edge X(1,*) which defines an undefined range. Finally, the patterns shown in Fig. 7e and f are examples of complex patterns containing multiple amino-acid-nodes, distance-edges, next-edges and gap-edges.

Table 4 contains the number of results obtained by searching the six examples described above. Note that each graphical representation maintains a clear similarity with its PROSITE textual representation.

This case study illustrates how starting from a very general search of Zn2+ binding sites with a certain sequence pattern different classes of Zinc-fingers can be identified in the entire PDB.

### Case study 3: exploration of structural patterns

Data exploration is about efficiently extracting knowledge from data even if we do not know exactly what we are looking for [30]. It usually implies to use visual exploration to understand what is in a dataset and the characteristics of the data. These characteristics can include size or amount of data, completeness of the data, correctness of the data, possible relationships amongst data elements, etc.

We used GSP4PDB to conduct a data exploration experiment to discover sub-patterns for a given pattern. In this case, we selected as target the Cys2His2 classical zinc finger, whose pattern is shown in Fig. 7a. Table 5 shows the exploration results.

**Table 5:** Summary of the results of case study 3: Sub-patterns of the Cys2His2 zinc finger

Gap			Protein			Cath Code (0 = No value)				AVG Distance			
G1	G2	G3	PDBs	Classification	Organism	C	A	T	H	ZN-Cys1	ZN-Cys2	ZN-His1	ZN-His2
2	12	2	4	TRANSCRIPTION (1),	HOMO SAPIENS (4)	0(2)	0(2)	0(2)	0(2)	2.25	2.31	2.07	5.49
				METAL BINDING		3(1)	30(1)	40(1)	200(1)				
				PROTEIN/DNA (1),		3(1)	30(1)	160(1)	60(1)				
				TRANSCRIPTION/DNA(1),									
				...									
2	12	3	4	TRANSCRIPTION (139),	HOMO SAPIENS (261),	0(198)	0(198)	0(198)	0(198)	2.33	2.26	2.08	2.07
				TRANSCRIPTION/DNA (91),	UNDEFINED (50),	2(1)	60(1)	40(1)	10(1)				
				GENE REGULATION/DNA (10),	MUS MUSCULUS (40),	3(172)	30(172)	160(172)	60(172)				
				UNKNOWN FUNCTION (5),	MUS (10),	3(4)	30(4)	428(4)	10(4)				
				...	...								
2	12	4	97	TRANSCRIPTION (29),	MUS MUSCULUS (6),	0(66)	0(66)	0(66)	0 (66)	2.30	2.24	2.05	2.05
				TRANSCRIPTION/DNA (22),	HOMO SAPIENS (70),	2(1)	170(1)	270(1)	10(1)				
				METAL BINDING PROTEIN (7),	UNDEFINED (11),	3(30)	30(30)	160(30)	60(30)				
				...	...								
2	12	5	15	TRANSCRIPTION (15),	HOMO SAPIENS (9),	0(4)	0(4)	0(4)	0(4)	2.32	2.29	2.37	2.60
				RNA BINDING	MUS MUSCULUS (1),	1(1)	10(1)	10(1)	790(1)				
				PROTEIN RNA (1),	XENOPUS LAEVIS (2),	3(10)	30(10)	160(10)	60(10)				
				...	...								
3	12	4	1	METAL BINDING PROTEIN (1)	HOMO SAPIENS (1)	0(1)	0(1)	0(1)	0(1)	6.00	1.72	1.94	2.32
3	12	5	2	RNA BINDING PROTEIN (69),	XENOPUS LAEVIS (1),	2(1)	30(1)	170(1)	10(1)	5.60	3.15	3.60	4.29
				METAL BINDING PROTEIN (1)	SYNECHOCOCCUS	3(1)	30(1)	160(1)	60(1)				
					ELONGATUS (1)								
4	12	3	117	TRANSCRIPTION DNA (69),	HOMO SAPIENS (63),	0(42)	0(42)	0(42)	0(42)	2.28	2.30	2.07	2.04
				TRANSCRIPTION	MUS MUSCULUS (19),	3(75)	30(75)	160(75)	60(75)				
				FACTOR/DNA (6),	ESCHERICHIA COLI (2),								
				...	...								
4	12	4	8	METAL BINDING PROTEIN (1),	HOMO SAPIENS (3),	0(4)	0(4)	0(4)	0(4)	2.26	2.26	2.07	2.14
				PROTEIN BINDING (1),	ARABIDOPSIS	3(4)	30(4)	160(4)	60(4)				
				DNA BINDING PROTEIN (2),	THALIANA (1),								
				...	...								
4	12	6	2	TRANSLATION	HOMO SAPIENS (2)	3(2)	30(2)	40(2)	130(2)	2.13	2.13	1.99	6.95
				REGULATOR (1),									
				METAL BINDING PROTEIN (1)									
			630							2.33	2.26	2.08	2.12

Each row contains information of a sub-pattern, where G1, G2 and G3 indicate the specific sizes for the gaps of the pattern shown in Fig. 7a. For instance, the textual representation of the first sub-pattern is C-X(2)-C-X(12)-H-X(2)-H

By using the filters of GSP4PDB, we were able to identify nine sub-patterns. Each sub-pattern is given by the specific sizes of the gaps (G1, G2, G3) defined by the main graph pattern. For instance, the first sub-pattern describes the structure C-X(2)-C-X(12)-H-X(2)-H. For each pattern, we group and show the number of PDBs, classifications, organisms, CATH codes and average distances. A number in parentheses is used to indicate the number of results for each specific value (“?” denotes an undefined value).

This structural bioinformatic search can be utilized as a starting point for further functional analysis of the sub-groups identified.

## Discussion

In this section we review related approaches, discuss the general use of graph theory in bioinformatics and discuss the advantages of the software presented here.

### Related work

We reviewed several tools related to visualize protein structures. Due to space restrictions we just mention a small number of tools and system. The following articles present reviews and comparisons of visualization tools: [31–33].

There are tools like JSMol [34], 3DMol [35], JSME [36], LigPlot+ [37], NGL [38] and ChemDoodle [39] which are oriented to visualize and edit molecular structures. These tools provide libraries for protein visualization which are used by databases and systems related to protein-ligand interactions. Among them we can mention Ligand Search (RCSB PDB), PDBbind [40], PDBSum [31], PubChem [41], AutoDock [42], iview [43], PDB-Ligand [44], Proteopedia [45] and sc-PDB [46]. In addition, there are a number of specialised graphical user interfaces designed for specific goals. For instance, PoseView [47] provides a special 2D diagram to visualize molecular interaction patterns, and GIANT [48] provides a 3D viewer based on density maps.

**Fig. 8:**
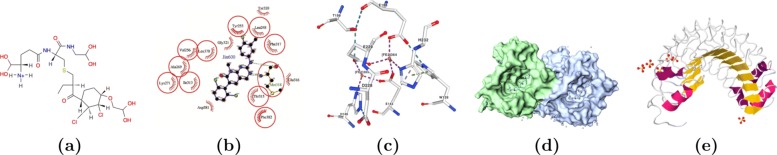
Types of charts used in protein structure visualization: (**a**) Molecular structure represented in JSME; (**b**) Interaction diagram used by LigPlot+; (**c**) Ball & Stick visualization provided by NGL (WebGL); (**d**) Surface representation supported by iview; (**e**) Cartoon visualization provided by JSmol

Figure 8 shows five types of graphical representations used to visualize distinct aspects of a protein’s structure. The molecular structure is usually represented by using a 2D schematic representation (Fig. 8a). The primary structure is visualized using chain-oriented representations (e.g. “wiring” diagrams), 2D charts (Fig. 8b) and 3D charts (Fig. 8c). A three-dimensional representation can use different types of shapes: lines, sticks, balls, spheres and surfaces (Fig. 8d). The secondary structure is also visualized in 3D, but using shapes like folds, strands, cylinders and plates (Fig 8e). The tertiary and quaternary structures are usually represented as a combination of 2D and 3D representations.

Our search and analysis of the related tools and systems, allowed us to verify that the graph-based representation of GSP4PDB is a novel approach. However, it is important to mention that most of the current approaches for visualizing protein structures share a common foundation: a graph-based structure.

In terms of interfaces for searching protein-ligand interactions, there are four basic types: form-based, text-based, sequence-based and molecular-based. A form-based interface [49] consists of a web form where the user is able to input different parameters (e.g. PDB code, protein name, ligand name, and so on) to conduct the search. In a text-based interface [40] a query is introduced as a textual representation (e.g. SMILES) of the protein-ligand interaction. A sequence-based interface [40] requires a textual representation (e.g. FASTA) of the protein sequence to conduct the search. A molecular-based interface [50] extends the schematic representation of a protein with complex conditional expressions for atoms and bonds. Most applications combine the above approaches to form a complex but multi-functional query and analysis interface.

In contrast to the current approaches, the graph-based query interface provided by GSP4PDB allows the user to create different types of structural patterns in a simple way (by doing drag-and-drop). Note that the notions of any-amino and any-ligand allows to explore and discover new kinds of structural patterns.

### Why a graph-based representation?

Graphs are omnipresent in our lives and have been increasingly used in a variety of application domains. In our context, graphs are a natural way of representing biological networks, and graph theoretical concepts are useful for the description and analysis of interactions and relationships in biological systems [51].

Different classes of graphs can be used to model different levels of abstraction and knowledge. For example, graphs have been used to represent protein-protein interaction networks [32] and cellular processes [52]. It is important to highlight that the graph-based model introduced in this article can be easily extended to support more complex protein structural patterns (e.g. patterns containing multiple ligands).

Protein data modeled as graphs is been supported by the development of graph database systems. These systems enable efficient storage and processing of the encoded biological relationships, and can offer great speedups over relational databases [53]. For instance, the Neo4j graph database (and its query language, Cypher) has been used for mining protein graphs [54], and to perform complex queries over biological pathway databases [55].

### Key features of gSP4PDB

Next we discuss the advantages of GSP4PDB in terms of the following features: multi-purpose, usability, efficiency, availability, maintainability, interoperability and multi-Platform.

**Multi-purpose** GSP4PDB allows the visualization, search and exploration of common structural patterns in protein-ligand interactions. Additionally, it facilitates the discovery of complex patterns that could be linked to the use or interaction with drugs or molecules of biotechnological interest. This information can be particularly important in the area of protein design and the creation of new enzymes.

**Usability** GSP4PDB provides a very simple and intuitive graph-based visual interface to represent a protein-ligand interaction. As discussed before, a graph is a powerful abstraction to represent the relationships between biological entities. The usability of GSP4PDB was evaluated by researchers and students of the bioinformatics department at Universidad de Talca (Chile).

**Efficiency** GSP4PDB allows fast and massive search of the structural characteristics in all the PDB data base facilitating the mining of data in three-dimensional information, something that is complex and expensive to do by traditional methods. The efficiency of the systems is given by the denormalized design of the database and the use of indexes. We believe that the efficiency could be improved with the use of a graph database system (instead of the current PostgreSQL relational database).

**Availability** GSP4PDB is hosted in a Lightsail virtual private server of Amazon Web Services (AWS). Hence, the system inherits AWS features like reliability, high-performance, persistent SSD-based block storage, load balancing, data protection and network stability.

**Maintainability** The installation of GSP4PDB in a web server is very simple and does not have special requirements. The most costly step (in terms of time) is the creation of the database due to the pre-processing of the entire PDB dataset. After the first data loading stage, the updating process is very simple thanks to the features of gsp4pdb-parser.

*Interoperability* A protein-ligand pattern can be stored as a JSON file, and can be loaded in the future. The results are shown by using either a traditional tabular representation or a graphical mode view (following a graph-based representation). The results can be exported as a text file with the list of protein IDs, or as a structured JSON file containing the encoding of the graph-based representation of each solution.

*Multi-platform* GSP4PDB is distributed as a web application and has been tested in all current browsers (see the section about Availability and requirements). The system does not require special plugins in the front-end, although requires PostgreSQL database system in the back-end.

## Conclusions

This paper presents GSP4PDB, a novel tool which allows to search protein-ligand interactions by using a simple and intuitive graphical representation based on graphs. Here, we describe the design and implementation of the three main elements, pre-processing, data storage and web interfaces. Furthermore, our case studies on the Zinc finger motifs demonstrate how new functionalities can be discovered for proteins with hitherto unknown function.

As future work we expect to extend the notion of protein-ligand structural patterns to support filters and advanced relationships (e.g. metal interaction geometries). Additionally, we will explore the use of big data technologies for storing and query PDB data. Particularly, we expect to use graph-based technologies like Giraph, a graph processing framework built on top of Apache Hadoop.

## Availability and requirements

**Project name**: GSP4PDB**Project home page**: http://gdblab.com/gsp4pdb/gsp4pdb2/**Hardware (host)**: Lightsail virtual private server, 2 Core Processor, 8 GB Memory, 160 GB SSD Disk, 5 TB Transfer**Operating system(s)**: Platform independent (Web)**Programming language**: PHP**Compatible Web navigators**: Chrome 71.0.3578.98, Internet Explorer 11, Opera 58.0.3135.47, Firefox 64.0.2, Safari 12.0.2**License**: Academic Free License (AFL)

## Data Availability

The datasets used and/or analyzed during the current study are available from the corresponding author on reasonable request and the PDB database.

## Abbreviations

ATP: Adenosine triphosphate
AWS: Amazon web services
DNA: Deoxyribonucleic acid
ID: Identifier
GSP: Graph-based structural pattern
PDB: Protein data bank
SMILES: Simplified molecular input line entry specification
